# Supplementary material for: Resolving the full spectrum of human genome variation using Linked-Reads
Source: Genome Res. 2019 Apr;29(4):635–45. doi: 10.1101/gr.234443.118 (PMC6442396; doi:10.1101/gr.234443.118)
Supplement: Supplemental Material [file supp_gr.234443.118_Supplemental_longranger-2.2.zip › longranger-2.2.tar.bz2!/lr2.2-public/lib/stan/cmdstan/stan/doc/api/html/classstan_1_1io_1_1cmd__line.html]

Stan: stan::io::cmd\_line Class Reference


|  |  |
| --- | --- |
|  | Stan  2.5.0  probability, sampling & optimization |


- Main Page
- Related Pages
- Namespaces
- Classes
- Files

- Class List
- Class Index
- Class Hierarchy
- Class Members

All Classes Namespaces Files Functions Variables Typedefs Enumerations Enumerator Friends Macros Pages

- stan
- io
- cmd\_line

Public Member Functions |
List of all members

stan::io::cmd\_line Class Reference

Parses and stores command-line arguments.
More...

`#include <cmd_line.hpp>`

|  |  |
| --- | --- |
| Public Member Functions | |
|  | cmd\_line (int argc, const char \*argv[]) |
|  | Construct a command-line argument object from the specified command-line arguments. More... |
|  | |
| std::string | command () |
|  | Returns the name of the command itself. More... |
|  | |
| bool | has\_key (const std::string &key) const |
|  | Return `true` if the specified key is defined. More... |
|  | |
| template<typename T > | |
| bool | val (const std::string &key, T &x) const |
|  | Returns the value for the key provided. More... |
|  | |
| bool | has\_flag (const std::string &flag) const |
|  | Return `true` if the specified flag is defined. More... |
|  | |
| size\_t | bare\_size () const |
|  | Return the number of bare arguments. More... |
|  | |
| template<typename T > | |
| bool | bare (size\_t n, T &x) const |
|  | Returns the bare argument. More... |
|  | |
| void | print (std::ostream &out) const |
|  | Print a human readable parsed form of the command-line arguments to the specified output stream. More... |
|  | |

## Detailed Description

Parses and stores command-line arguments.

Command-line arguments are organized into four types.

**Command**: The first argument (at index 0) is just the command itself. There method `command()` retrieves the command.

**Key/Value**: The second type of argument is a key-value pair, which must be in the form `–key=val`. Two hyphens are used to separate arguments from negated numbers. The method `has_key(const std::string&)` indicates if there is a key and `val(const std::string&,T&)` writes its value into a reference (whose type is templated; any type understand by the output operator `>>` is acceptable.

**Flag**: Flags are specified as `–flag`. The method `has_flag(const std::string&)` tests if a flag is present.

**Bare Argument**: Bare arguments are any arguments that are not prefixed with two hyphens (`–`). The method `bare_size()` returns the number of bare arguments and they are retrieved with the generic method `bare(const std::string&,T&)`.

Definition at line 113 of file cmd\_line.hpp.

## Constructor & Destructor Documentation

|  |  |  |  |
| --- | --- | --- | --- |
| stan::io::cmd\_line::cmd\_line | ( | int | *argc*, |
|  |  | const char \* | *argv*[] |
|  | ) |  |  |

inline

Construct a command-line argument object from the specified command-line arguments.

Parameters
:   |  |  |
    | --- | --- |
    | argc | Number of arguments. |
    | argv | Argument strings. |

Definition at line 142 of file cmd\_line.hpp.

## Member Function Documentation

template<typename T >

|  |  |  |  |
| --- | --- | --- | --- |
| bool stan::io::cmd\_line::bare | ( | size\_t | *n*, |
|  |  | T & | *x* |
|  | ) |  | const |

inline

Returns the bare argument.

If the specified index is valid for bare arguments, write the bare argument at the specified index into the specified reference, and otherwise return false without modifying the reference.

Parameters
:   |  |  |  |
    | --- | --- | --- |
    | [in] | n | Bare argument position. |
    | [out] | x | Reference to result. |

Returns
:   `true` if there were enough bare arguments.

Template Parameters
:   |  |  |
    | --- | --- |
    | T | Type of value returned. |

Definition at line 230 of file cmd\_line.hpp.

|  |  |  |  |  |
| --- | --- | --- | --- | --- |
| size\_t stan::io::cmd\_line::bare\_size | ( |  | ) | const |

inline

Return the number of bare arguments.

Returns
:   Number of bare arguments.

Definition at line 212 of file cmd\_line.hpp.

|  |  |  |  |  |
| --- | --- | --- | --- | --- |
| std::string stan::io::cmd\_line::command | ( |  | ) |  |

inline

Returns the name of the command itself.

The command is always supplied as the first argument (at index 0).

Returns
:   Name of command.

Definition at line 155 of file cmd\_line.hpp.

|  |  |  |  |  |  |
| --- | --- | --- | --- | --- | --- |
| bool stan::io::cmd\_line::has\_flag | ( | const std::string & | *flag* | ) | const |

inline

Return `true` if the specified flag is defined.

Parameters
:   |  |  |
    | --- | --- |
    | flag | Flag to test. |

Returns
:   `true` if flag is defined.

Definition at line 203 of file cmd\_line.hpp.

|  |  |  |  |  |  |
| --- | --- | --- | --- | --- | --- |
| bool stan::io::cmd\_line::has\_key | ( | const std::string & | *key* | ) | const |

inline

Return `true` if the specified key is defined.

Parameters
:   |  |  |
    | --- | --- |
    | key | Key to test. |

Returns
:   `true` if it has a value.

Definition at line 165 of file cmd\_line.hpp.

|  |  |  |  |  |  |
| --- | --- | --- | --- | --- | --- |
| void stan::io::cmd\_line::print | ( | std::ostream & | *out* | ) | const |

inline

Print a human readable parsed form of the command-line arguments to the specified output stream.

Parameters
:   |  |  |  |
    | --- | --- | --- |
    | [out] | out | Output stream. |

Definition at line 245 of file cmd\_line.hpp.

template<typename T >

|  |  |  |  |
| --- | --- | --- | --- |
| bool stan::io::cmd\_line::val | ( | const std::string & | *key*, |
|  |  | T & | *x* |
|  | ) |  | const |

inline

Returns the value for the key provided.

If the specified key is defined, write the value of the key into the specified reference and return `true`, otherwise do not modify the reference and return `false`.

The conversions defined by `std::ostream` are used to convert the base string value to the specified type. Thus this method will work as long as `operator>>()` is defined for the specified type.

Parameters
:   |  |  |  |
    | --- | --- | --- |
    | [in] | key | Key whose value is returned. |
    | [out] | x | Reference to value. |

Returns
:   False if the key is not found, and true if it is found.

Template Parameters
:   |  |  |
    | --- | --- |
    | Type | of value. |

Definition at line 189 of file cmd\_line.hpp.

---

The documentation for this class was generated from the following file:

- src/stan/io/cmd\_line.hpp


---

[ Stan Home Page ]

*© 2011–2014,
Stan Development Team.*
